# Association between fish oil and glucosamine use and mortality in patients diagnosed with cancer: the role of the Life Essential 8 score and cancer prognosis

**DOI:** 10.1186/s12937-024-01032-1

**Published:** 2024-10-17

**Authors:** Chun Sing Lam, Rong Hua, Herbert Ho-Fung Loong, Vincent Chi-Ho Chung, Yin Ting Cheung

**Affiliations:** 1 School of Pharmacy, Faculty of Medicine, The Chinese University of Hong Kong, Hong Kong, China; 2 Department of Clinical Oncology, Faculty of Medicine, The Chinese University of Hong Kong, Hong Kong, China; 3 Jockey Club School of Public Health and Primary Care, Faculty of Medicine, The Chinese University of Hong Kong, Hong Kong, China; 4 Hong Kong Hub of Pediatric Excellence, The Chinese University of Hong Kong, Hong Kong, China

**Keywords:** Fish oil, Glucosamine, Mortality, Prognosis, Cancer patients, Life Essential 8

## Abstract

**Background:**

The effect of supplements on mortality risk in patients with cancer remains uncertain and has scarcely been investigated in subgroups of patients with varying characteristics. This study aimed to investigate the association between two popular supplements, fish oil and glucosamine, and mortality risk in a large population-based cohort and determine whether cardiovascular health and clinical prognosis influence these associations.

**Methods:**

This prospective cohort study analyzed the data of UK Biobank participants who were diagnosed with cancer. The associations of fish oil and glucosamine consumption with mortality were analyzed using Cox proportional hazards models. Subgroup analyses were performed to assess the effects of Life Essential 8 [LE8] scores (a measure of cardiovascular health) and cancer prognosis (grouped according to the survival rates of specific cancer types) on the associations between supplement use and mortality.

**Results:**

This analysis included 14,920 participants (mean age = 59.9 years; 60.2% female). One third (34.1%) of the participants reported using fish oil, and one fifth (20.5%) reported using glucosamine. Over a median follow-up of 12.0 years, 2,708 all-cause deaths were registered. The use of fish oil was associated with reduced risks of all-cause mortality (adjusted hazard ratio [aHR] = 0.89, 95% Confidence Interval [CI] = 0.81–0.97) and cancer mortality (aHR = 0.89, 95% CI = 0.81–0.98). Similarly, glucosamine use was associated with reduced risks of all-cause mortality (aHR = 0.83, 95% CI = 0.74–0.92) and cancer mortality (aHR = 0.83, 95% CI = 0.74–0.93) in the fully adjusted model. Subgroup analyses revealed that the protective effects of fish oil and glucosamine against mortality risk were only observed in patients with LE8 scores lower than the mean score or a poor cancer prognosis. Additionally, the association between glucosamine use and a reduced risk of CVD-related mortality was only observed in patients with lower LE8 scores.

**Conclusions:**

This large cohort study identified the potential differential impact of LE8 scores and cancer prognosis on the associations of fish oil and glucosamine supplementation with survival in patients with cancer. This suggests the importance of considering these factors in future research on supplements and in the provision of personalized integrative cancer care.

**Supplementary Information:**

The online version contains supplementary material available at 10.1186/s12937-024-01032-1.

## Background

Patients with cancer often use herbal and dietary supplements (HDS), such as vitamins, minerals, and plant-based supplements. The prevalence of HDS use ranged from 33 to 81% in cancer populations of the United States (US) and the United Kingdom (UK) [1–5]. Many patients with cancer use HDS to reduce the risk of cancer recurrence, improve their immune system, promote general well-being, or manage cancer-related symptoms [4–7]. Despite their widespread popularity, the evidence for the association between specific dietary supplements and mortality in patients with cancer remains conflicting and inconclusive [8–14].

The majority of studies have largely focused on the use of vitamin and mineral supplements by patients with cancer [9–12, 14, 15]. However, patients with cancer also frequently use other non-vitamin, non-mineral dietary supplements [3, 4, 16, 17]. Among the non-vitamin, non-mineral supplements, fish oil and glucosamine are reported to be commonly used by this patient population [3, 4, 16]. The associations between their use and mortality in the general populations have been studied in few large-scale studies [18–21]. These studies have found the use of glucosamine and fish oil to be associated with a reduced risk of mortality [18–21]. The mechanism through which fish oil and glucosamine are associated with a lower risk of mortality remains unclear. One potential mechanism could be linked to their impact on inflammation, which plays an important role in cancer development, progression, and prognosis [22]. Higher levels of inflammation have been shown to potentially increase the risks of all-cause and cancer mortality [23, 24]. Fish oil contains omega-3 fatty acids (docosahexaenoic acid [DHA] and eicosapentaenoic acid [EPA]). Both glucosamine and omega-3 may potentially reduce inflammation through various pathways, such as suppressing inflammatory cell activation or promoting noninflammatory efferocytosis [25, 26]. Another potential mechanism regarding glucosamine may involve its ability to mimic the effects of a low carbohydrate diet [27]. Several large cohort studies have shown the protective effect of a low carbohydrate diet on mortality [28, 29]. However, studies focusing on the effects of these supplements on mortality in the cancer patient population are currently lacking.

It is recognized that supplement users tend to have healthier lifestyles, including engaging in regular exercise and having healthier dietary patterns with a lower metabolic risk, than non-users [30, 31]. Some studies examining the associations of fish oil and glucosamine consumption with mortality in the general population have revealed that a lower risk of mortality may be correlated with certain health characteristics, such as smoking, cholesterol levels, and obesity [18, 20]. Therefore, adjustment or stratification based on these characteristics should be considered when examining the effects of supplements [31, 32]. A combination of health metrics, the Life Essential 8 (LE8) score, has been defined by the American Heart Association [33]. The score is assigned based on a participant’s adherence to eight healthy lifestyle components aimed at improving and maintaining cardiovascular health and reducing the risk of heart diseases and other major health problems. A higher LE8 score has been linked to lower mortality in both cancer and non-cancer populations [34]. While some studies have suggested potential benefits of dietary supplements for reducing mortality, it remains unknown whether these benefits hold true for all patients with cancer [35]. There has been extensive research on the role of fish oil in cardiovascular health, whereas only preliminary evidence is available for glucosamine [36–38]. Studies specifically focused on the cancer population are currently lacking. Fish oil (EPA + DHA or EPA-only) is indicated for reducing triglycerides, and is considered a reasonable option for secondary prevention in patients with recent coronary heart disease events or heart failure, according to the guidelines by the American Heart Association [38]. However, evidence regarding the effects of fish oil and glucosamine in lowering the risk of cardiovascular events remains inconclusive, although some studies suggested potential benefits [36, 39]. Multiple mechanisms, such as antithrombotic and anti-inflammatory effects, may contribute to their potential roles in cardiovascular health [26, 37]. In terms of cancer prognosis, little is known about the anti-cancer effects of these supplements and their direct impact on cancer prognoses, as most evidence comes from *in-vitro* studies [40, 41]. Both fish oil and glucosamine are known to reduce inflammation, and anti-inflammatory properties may potentially improve cancer prognosis [42, 43]. Supplements may also improve clinical prognosis through indirect pathways, for example, fish oil ameliorates cancer cachexia, which is linked to poor cancer prognosis [44]. It remains unclear whether the benefits of these supplements are similar across patients diagnosed with cancers of different prognoses.

The objectives of this study were to (1) examine the associations between the use of fish oil and glucosamine and all-cause, cancer, and cardiovascular disease (CVD)-related mortality in patients with cancer, and (2) explore any differences in these associations among subgroups of patients with LE8 scores lower or higher than the mean score, as well as patients with a good or poor cancer prognosis, in a large population-based cohort in the UK.

## Methods

This study was registered with the UK Biobank (ref.: 74158) and is reported according to the STROBE (STrengthening the Reporting of OBservational studies in Epidemiology) guidelines [45]. The UK Biobank study was approved by the National Health Service North West Multi-centre Research Ethics Committee and the participants provided written informed consent [46].

### Study population

The UK Biobank is a large population-based cohort of approximately 500,000 participants in the UK. It facilitates investigation of a wide range of complex diseases of middle- and old-age individuals [47]. The UK Biobank study first recruited participants aged 40–69 years across the UK during 2006 to 2010 and has since conducted repeated assessments. The methods used have been reported in detail elsewhere [47].

In this study, participants were excluded if 1) they had not been diagnosed with malignant cancer prior to baseline recruitment or 2) they had not provided responses to questions related to the use of dietary supplements, any components of the LE8 score, or any other covariates.

### Cancer diagnosis and prognosis based on cancer types

The UK Biobank is linked to national cancer registries (Health and Social Care Information Centre and the National Health Service Central Register) [48]. Cancer diagnoses were coded according to the International Classification of Diseases, 9th and 10th revisions (ICD-9 and ICD-10). We included only malignant neoplasms (ICD-9: 140–208; ICD-10: C00–C97), except non-melanoma skin cancer (ICD-9: 173; ICD-10: C44), in this study. Regarding cancer prognoses, the types of cancer were categorized into two groups (good versus poor) according to the UK statistics on the survival rates of patients with cancer [49, 50], as well as mortality rates of the cancers in the UK Biobank sample (details of classification in Supplementary Table 1). This classification approach is consistent with other studies analysing cancer-related outcomes in large population-based databases which cancer staging or subtypes data are not available [51, 52].

### Ascertainment of the use of fish oil and glucosamine

The participants were asked, “Do you regularly take any of the following?” and were provided with a list of supplements, including fish oil (such as cod liver oil) and glucosamine, in a touchscreen questionnaire. The participants who did not select either fish oil or glucosamine or indicated “None of the above” were considered non-users of these supplements.

### LE8 score calculation

The LE8 score consists of eight components related to health behaviors and factors, namely smoking, sleep, diet, exercise, blood pressure, blood lipids, blood glucose, and body mass index (BMI) [33]. Information on smoking status, physical activity, sleep, dietary intake, and BMI was collected using a touchscreen questionnaire at baseline. Quantitative measurements, namely blood pressure, non-high-density lipoprotein (HDL) cholesterol concentrations, and glycated hemoglobin (HbA1c) concentrations, were conducted during the baseline assessment. In this study, a modified version of the LE8 score was adopted based on previous studies that used the UK Biobank data [53]. The classification of the LE8 score components followed the original version, except that the dietary pattern was evaluated using a previous dietary score adapted for the UK Biobank data instead of the DASH-style eating pattern [53, 54]. Detailed information on the LE8 score evaluation is presented in Supplementary Table 2. The score for each component ranges from 0 (the least healthy) to 100 (the healthiest). The overall LE8 score is calculated by dividing the sum of the scores for all eight components by 8.

### Ascertainment of mortality outcomes

The UK Biobank obtained comprehensive mortality data (the date and cause of death) from the Information Centre (England and Wales) and the National Health Service Central Register Scotland [47]. ICD-10 codes were used in the death records to identify the causes of death (primary and contributory). In the current study, all-cause mortality and mortality due to cancer (C00–C97) and CVDs (I00–I99) were analyzed. The participants were followed up from the date of recruitment (2006–2010) until the date of death or the end of the follow-up period (March 23, 2021, or earlier if they were lost to follow-up), whichever occurred first.

### Assessment of confounders

Potential confounders commonly associated with the use of fish oil and glucosamine and mortality in patients with cancer were selected *a priori* based on data from the literature [55–60]. These were sociodemographic factors (sex, age, Townsend Deprivation Index score, and educational level), alcohol consumption, oily fish consumption, and the use of vitamin or mineral supplements, which were collected using the touchscreen questionnaire at baseline. Clinical confounders were the time since cancer diagnosis, CVD diagnosis prior to cancer diagnosis, and other comorbidities [57, 59]. The Charlson Comorbidity Index (CCI) was used to quantify the comorbidity burden of the participants prior to their cancer diagnosis [61]. CVD diagnoses (namely ischemic heart diseases, myocardial infarction, stroke, and heart failure) were ascertained from linked hospitalization and death records using ICD-10 codes: I20–25, I50, I60–64, and I70–74. Vitamin or mineral supplement users were defined as those who selected any of the vitamin or mineral supplements mentioned in the touchscreen questionnaire. Detail information regarding the confounders are presented in Supplementary Table 1.

### Statistical analyses

Descriptive statistics were used to summarize the baseline characteristics of the participants. The associations between the use of fish oil and glucosamine and the risks of all-cause and cause-specific mortality were analyzed using Cox proportional hazards models. The proportional hazard assumption was assessed using tests based on Schoenfeld residuals [62]. No violation of the assumption was detected in the analyses. Three models were run: a crude model (Model 1), an age- and sex-adjusted model (Model 2), and a model fully adjusted for factors identified a priori, which are age, sex, socioeconomic factors, the LE8 score and other lifestyle factors, time since cancer diagnosis, the CCI score, CVD diagnosis prior to assessment, cancer prognosis (good versus poor), and vitamin or mineral supplement use (Model 3). The analysis compared participants who regularly used fish oil with non-users, as well as those who regularly used glucosamine with non-users. In the analysis of the combined use of fish oil and glucosamine, the reference group consisted of participants who reported regular use of neither fish oil nor glucosamine. The index date was defined as the date of baseline assessment. A competing risk analysis was performed to measure the associations between supplement use and cause-specific mortality while considering death due to causes other than the studied cause as competing risks.

Subgroup analyses were performed by dividing the included patients according to their LE8 scores (above or below the mean score) and cancer prognoses (good versus poor). The interactions between the use of fish oil or glucosamine and LE8 scores, as well as between the use of fish oil or glucosamine and cancer prognosis, were tested using a likelihood ratio test comparing models with and without a cross-product term (for multiplicative interactions) and relative excess risk due to interaction (RERI) (for additive interactions) [63, 64]. To assess the joint effect, participants were classified into four groups based on their regular supplement use status and either their LE8 scores (above or below mean) or their cancer prognoses (good or poor). Hazard ratios (HR) were estimated to compare these groups with participants who did not regularly use fish oil or glucosamine and had a lower LE8 scores (mean or below), or who had cancers of poor prognosis.

Two sensitivity analyses were performed. First, deaths within 2 years after baseline assessments were excluded to avoid reverse causality bias. Second, the analyses of the associations between the use of fish oil or glucosamine and cause-specific mortality were repeated without considering competing risk. All of the statistical analyses were performed using R version 4.0.3. A *p*-value < 0.05 was considered statistically significant.

## Results

### Baseline characteristics

**Fig. 1:**
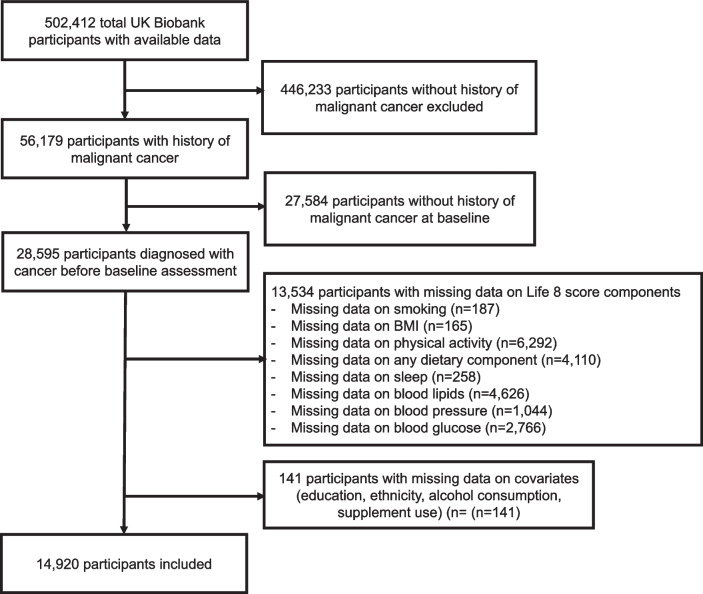
Flowchart of participants inclusion

Of the total 502,412 participants in the UK Biobank cohort, participants were excluded if 1) they had not been diagnosed with malignant cancer prior to baseline recruitment (*n* = 473,817) or 2) data on any of their sociodemographic or lifestyle factors were missing (for LE8 score computations) (*n* = 13,675). Finally, 14,920 participants were included in the analysis (Fig. 1).

The mean age of the participants at recruitment in the UK Biobank study was 59.9 (SD = 7.1) years, and 60.2% of them were female (Tables 1 and 2, Supplementary Table 3). The mean age at first cancer diagnosis was 52.4 (SD = 10.3) years, and the median time since cancer diagnosis was 6.0 (interquartile range [IQR] = 2.0–11.0) years. The most common cancer diagnoses were breast (*n* = 4,889, 32.8%) and genitourinary (*n* = 3,073, 20.6%) cancers. About 30% of the participants (*n* = 4,691) were diagnosed with cancers with poor prognosis. One third of the participants (*n* = 5,093, 34.1%) reported the use of fish oil, and one fifth (*n* = 3,066, 20.5%) reported the use of glucosamine.

**Table 1:** Baseline characteristics of participants (classified by fish oil use status) (*N* = 14,920)

	**Fish oil users (*n* = 5,093)**	**%**	**Fish oil non-users (*n* = 9,827)**	**%**	*P*^*a*^
**Sociodemographics**					
**Sex**					
Male	1,959	38.5	3,981	40.5	**0.016**
Female	3,134	61.5	5,846	59.5	
**Age attending assessment centres (Mean ± SD)**	61.4	± 6.2	59.2	± 7.4	** < 0.001**
**Townsend deprivation index (Mean ± SD)**	-1.7	± 2.9	-1.5	± 3.0	** < 0.001**
**Ethnic background**					
White	4,978	97.7	9,579	97.5	0.346
Others	115	2.3	248	2.5	
**Education**					
College or university degree	1,580	31.0	3,259	33.2	**0.009**
Below degree	3,513	69.0	6,568	66.8	
**Life Essential 8 scores**					
Total score (Mean ± SD)	65.1	± 11.4	63.5	± 12.3	** < 0.001**
BMI score (Mean ± SD)	71.5	± 26.7	69.3	± 28.7	** < 0.001**
Nicotine exposure score (Mean ± SD)	72.1	± 30.9	71.1	± 32.7	** < 0.001**
Physical activity score (Mean ± SD)	78.3	± 35.4	72.6	± 38.7	** < 0.001**
Sleep health score (Mean ± SD)	89.7	± 18.3	88.7	± 19.5	** < 0.001**
Diet score (Mean ± SD)	37.5	± 28.5	32.1	± 28.3	** < 0.001**
Blood lipids score (Mean ± SD)	46.0	± 28.4	48.0	± 29.3	** < 0.001**
Blood glucose score (Mean ± SD)	90.3	± 19.3	89.3	± 20.8	** < 0.001**
Blood pressure score (Mean ± SD)	35.1	± 26.9	37.0	± 27.0	** < 0.001**
**Lifestyle**					
**BMI (Mean ± SD)**	27.0	± 4.3	27.3	± 4.8	** < 0.001**
**Smoking status**					
Never	2,592	50.9	5,055	51.5	** < 0.001**
Former	2,156	42.3	3,865	39.3	
Current	345	6.8	907	9.2	
**Alcohol consumption**					
Never	174	3.4	405	4.1	**0.026**
Former	202	4.0	445	4.5	
Current	4,717	92.6	8,977	91.4	
**Vitamin or mineral supplement use**	3,365	66.1	3,008	30.6	** < 0.001**
**Oily fish consumption (≥ 2 servings/week)**	3,483	68.4	5,786	58.9	** < 0.001**
**Clinical**					
**Prior cardiovascular diseases**	374	7.3	752	7.7	**0.025**
**Cancer diagnoses (Top 5)**					
Breast	1,739	34.1	3,150	32.1	**0.013**
Genitourinary	1,114	21.9	1,959	20.0	**0.010**
Digestive organs/Gastrointestinal	505	9.9	1,199	12.2	** < 0.001**
Melanoma	466	9.1	836	8.5	0.197
Hematological	382	7.5	869	8.8	**0.010**
**Class of cancer diagnoses (ICD codes)**					
Lip, oral cavity and pharynx (C00-14)	94	1.8	214	2.2	0.395
Digestive organs (C15-26)	505	9.9	1,199	12.2	** < 0.001**
Respiratory and intrathoracic organs (C30-39)	104	2.0	215	2.2	0.823
Bone and articular cartilage (C40-41)	22	0.4	41	0.4	1
Malignant melanoma of skin (C43)	466	9.1	836	8.5	0.395
Mesothelial and soft tissue (C45-49)	54	1.1	95	1.0	0.823
Breast (C50)	1,739	34.1	3,150	32.1	**0.037**
Female genital organs (C51-58)	471	9.2	906	9.2	1
Male genital organs (C60-63)	896	17.6	1,519	15.5	**0.006**
Urinary tract (C64-68)	219	4.3	442	4.5	0.823
Eye, brain and other parts of CNS (C69-72)	49	1.0	113	1.1	0.584
Thyroid and other endocrine glands (C73-75)	74	1.5	183	1.9	0.222
Ill-defined, secondary and unspecified sites (C76-80)	44	0.9	89	0.9	1
Primary, of lymphoid, haematopoietic and related tissue (C81-96)	382	7.5	869	8.8	**0.026**
**Age at first cancer diagnosis (Mean ± SD)**	53.5	± 9.9	51.9	± 10.4	** < 0.001**
**Year since cancer diagnosis (Median [IQR))**	6.0	2.0–12.0	5.0	2.0–11.0	** < 0.001**

*Abbreviation CNS* Central nervous system

^a^Characteristics between supplement users and non-users were compared using the Chi-square test, t-test, or Wilcoxon rank sum test. False discovery rate-adjusted P-values were computed to account for multiple testing

**Table 2:** Baseline characteristics of participants (classified by glucosamine use status) (*N* = 14,920)

	**Glucosamine users (*n* = 3,066)**	**%**	**Glucosamine non-users (*n* = 11,854)**	**%**	***P***^a^
**Sociodemographics**					
**Sex**					
Male	978	31.9	4,962	41.9	** < 0.001**
Female	2,088	68.1	6,892	58.1	
**Age attending assessment centres (Mean ± SD)**	61.5	± 5.9	59.5	± 7.3	** < 0.001**
**Townsend deprivation index (Mean ± SD)**	-1.9	± 2.7	-1.5	± 3.0	** < 0.001**
**Ethnic background**					
White	2,998	97.8	11,559	97.5	0.423
Others	68	2.2	295	2.5	
**Education**					
College or university degree	1,030	33.6	3,809	32.1	0.129
Below degree	2,036	66.4	8,045	67.9	
**Life Essential 8 scores**					
Total score (Mean ± SD)	65.6	± 11.5	63.6	± 12.2	** < 0.001**
BMI score (Mean ± SD)	70.5	± 27.5	69.9	± 28.2	** < 0.001**
Nicotine exposure score (Mean ± SD)	73.0	± 30.0	71.0	± 32.6	** < 0.001**
Physical activity score (Mean ± SD)	79.6	± 34.5	73.3	± 38.4	** < 0.001**
Sleep health score (Mean ± SD)	90.1	± 17.8	88.7	± 19.4	** < 0.001**
Diet score (Mean ± SD)	38.6	± 29.1	32.7	± 28.2	** < 0.001**
Blood lipids score (Mean ± SD)	45.7	± 28.8	47.7	± 29.1	** < 0.001**
Blood glucose score (Mean ± SD)	90.8	± 18.8	89.3	± 20.6	** < 0.001**
Blood pressure score (Mean ± SD)	36.3	± 26.7	36.4	± 27.0	** < 0.001**
**Lifestyle**					
**BMI (Mean ± SD)**	27.1	± 4.3	27.2	± 4.7	** < 0.001**
**Smoking status**					
Never	1,582	51.6	6,065	51.2	** < 0.001**
Former	1,314	42.9	4,707	39.7	
Current	170	5.5	1,082	9.1	
**Alcohol consumption**					
Never	83	2.7	496	4.2	** < 0.001**
Former	106	3.5	541	4.6	
Current	2,877	93.8	10,817	91.2	
**Vitamin or mineral supplement use**	1,991	64.9	4,382	37.0	** < 0.001**
**Clinical**					
**Prior cardiovascular diseases**	158	5.2	968	8.2	** < 0.001**
**Cancer diagnoses (Top 5)**					
Breast	1,197	39.0	3,692	31.1	** < 0.001**
Genitourinary	585	19.1	2,488	21.0	**0.027**
Digestive organs/Gastrointestinal	284	9.3	1,420	12.0	** < 0.001**
Melanoma	280	9.1	1,022	8.6	0.391
Hematological	200	6.5	1,051	8.9	** < 0.001**
**Class of cancer diagnoses (ICD codes)**					
Lip, oral cavity and pharynx (C00-14)	54	1.8	254	2.1	0.294
Digestive organs (C15-26)	284	9.3	1,420	12.0	** < 0.001**
Respiratory and intrathoracic organs (C30-39)	44	1.4	275	2.3	**0.011**
Bone and articular cartilage (C40-41)	18	0.6	45	0.4	0.241
Malignant melanoma of skin (C43)	280	9.1	1,022	8.6	0.409
Mesothelial and soft tissue (C45-49)	26	0.8	123	1.0	0.409
Breast (C50)	1,197	39.0	3,692	31.1	** < 0.001**
Female genital organs (C51-58)	308	10.0	1,069	9.0	0.150
Male genital organs (C60-63)	461	15.0	1,954	16.5	0.112
Urinary tract (C64-68)	124	4.0	537	4.5	0.337
Eye, brain and other parts of CNS (C69-72)	22	0.7	140	1.2	0.081
Thyroid and other endocrine glands (C73-75)	36	1.2	221	1.9	**0.031**
Ill-defined, secondary and unspecified sites (C76-80)	23	0.8	110	0.9	0.409
Primary, of lymphoid, haematopoietic and related tissue (C81-96)	200	6.5	1,051	8.9	** < 0.001**
**Age at first cancer diagnosis (Mean ± SD)**	53.6	± 10.4	52.1	± 9.7	** < 0.001**
**Year since cancer diagnosis (Median [IQR))**	6.0	2.0–12.0	5.0	2.0–11.0	** < 0.001**

*Abbreviation CNS* Central nervous system

^a^Characteristics between supplement users and non-users were compared using the Chi-square test, t-test, or Wilcoxon rank sum test. False discovery rate-adjusted *P*-values were computed to account for multiple testing

The mean LE8 score among the included participants was 64.0 (SD = 12.1) and was similar among fish oil and glucosamine users and non-users. Regarding the individual components, the mean score ranged from 33.9 (diet score) to 89.6 (blood glucose score).

### Associations of fish oil and glucosamine use with all-cause, cancer, and CVD-related mortality

At a median follow-up of 12.0 (IQR = 11.1–12.8) years, 2,708 all-cause deaths, 2,204 cancer deaths, and 590 CVD-related deaths were registered. The use of fish oil was associated with reduced risks of all-cause mortality (adjusted hazard ratio [aHR] = 0.89, 95% confidence interval [CI] = 0.81–0.97, *p* = 0.005) and cancer mortality (aHR = 0.89, 95% CI = 0.81–0.98,* p* = 0.023), but not CVD-related mortality, in the fully adjusted model (Table 3). Similarly, glucosamine use was associated with reduced risks of all-cause mortality (aHR = 0.83, 95% CI = 0.74–0.92, *p* < 0.001) and cancer mortality (aHR = 0.83, 95% CI = 0.74–0.93, *p* = 0.001) in the fully adjusted model. The use of glucosamine was also associated with a reduced risk of CVD-related mortality in the age- and sex-adjusted model (aHR = 0.66, 95% CI = 0.53–0.83, *p* < 0.001) but not in the fully adjusted model (aHR = 0.81, 95% CI = 0.64–1.02, *p* = 0.098).

**Table 3:** Associations of fish oil and glucosamine use with risks of overall, cancer and CVD mortality

	**Death among users**	**Death among non-users**	**Model 1 (Crude)**		**Model 2 **^**a**^		**Model 3 **^**a**^	
	N (%)	N (%)	Hazard ratio (95% CI)	*P*	Hazard ratio (95% CI)	*P*	Hazard ratio (95% CI)	*P*
**Fish oil**								
All-cause mortality	866 (17.0)	1,838 (18.7)	0.89 (0.82–0.97)	**0.006**	0.83 (0.76–0.90)	** < 0.001**	0.89 (0.81–0.97)	**0.005**
Cancer mortality	696 (13.7)	1,508 (15.3)	0.88 (0.80–0.96)	**0.005**	0.83 (0.76–0.91)	** < 0.001**	0.89 (0.81–0.98)	**0.023**
CVD mortality	203 (4.0)	387 (3.9)	1.01 (0.85–1.19)	0.930	0.90 (0.76–1.06)	0.220	1.02 (0.85–1.22)	0.850
**Glucosamine**								
All-cause mortality	456 (14.9)	2,248 (19.0)	0.77 (0.69–0.85)	** < 0.001**	0.75 (0.67–0.83)	** < 0.001**	0.83 (0.74–0.92)	** < 0.001**
Cancer mortality	370 (12.1)	1,834 (15.5)	0.77 (0.69–0.86)	** < 0.001**	0.75 (0.67–0.84)	** < 0.001**	0.83 (0.74–0.93)	**0.001**
CVD mortality	88 (2.9)	502 (4.2)	0.68 (0.54–0.85)	**0.001**	0.66 (0.53–0.83)	** < 0.001**	0.81 (0.64–1.02)	0.098

^a^Model 2: adjusted for age and sex; Model 3: adjusted for age, sex, ethnicities, socio-economic (Townsend deprivation index score and education level), Life 8 essential scores, alcohol status, time since cancer diagnosis, CVD diagnosis prior to assessment, vitamin or mineral supplement use, Charlson comorbidity index, cancer prognosis, oily fish consumption (only for fish oil)

The results of our sensitivity analyses of the associations between the use of fish oil or glucosamine and mortality were mostly consistent with the results of the main analyses (Supplementary Table 4). The combined use of fish oil and glucosamine and mortality was also found to be associated with reduced risks of all-cause mortality and cancer mortality (both *p* < 0.005), but not CVD-related mortality, in the fully adjusted model (Supplementary Table 5).

### Associations of fish oil and glucosamine use with mortality in participants with lower versus higher LE8 scores

When stratified into two groups by the mean LE8 score, associations between the use of fish oil or glucosamine and a reduced risk of mortality were only observed in the group with LE8 scores lower than the mean score (Table 4). Among participants with lower LE8 scores, the use of fish oil was associated with a reduced risk of all-cause mortality (aHR = 0.87, 95% CI = 0.78–0.97, *p* = 0.016) and a marginal reduction in the risk of cancer mortality (aHR = 0.88, 95% CI = 0.79–1.00, *p* = 0.049). Similarly, significant inverse associations were found between glucosamine use and all-cause (aHR = 0.69, 95% CI = 0.60–0.80, *p* < 0.001), cancer (aHR = 0.71, 95% CI = 0.61–0.84, *p* < 0.001), and CVD-related (aHR = 0.71, 95% CI = 0.52–0.97, *p* = 0.030) mortality in this group of participants. No significant associations were observed between the use of fish oil or glucosamine and the risk of mortality in the group of participants with higher LE8 scores. The results regarding the combined use of fish oil and glucosamine were consistent with those of the individual supplements (Supplementary Table 5).

**Table 4:** Associations of fish oil and glucosamine use with risks of overall, cancer and CVD mortality stratified by Life 8 Essential Score class

	**Death among users**	**Death among non-users**	**Class 1 (Mean score or below) (*n* = 7,492)**		**Death among users**	**Death among non-users**	**Class 2 (Above mean score) (*n* = 7,428)**	
	N (%)	N (%)	Hazard ratio (95% CI)	*P*			Hazard ratio (95% CI)	*P*
**Fish oil**								
All-cause mortality	497 (20.7)	1,168 (23.0)	0.87 (0.78–0.97)	**0.016**	369 (13.7)	670 (14.1)	0.89 (0.77–1.02)	0.081
Cancer mortality	386 (16.1)	923 (18.1)	0.88 (0.79–1.00)	**0.049**	310 (11.5)	585 (12.3)	0.87 (0.76–1.01)	0.074
CVD mortality	134 (5.6)	282 (5.5)	0.98 (0.78–1.23)	0.860	69 (2.6)	105 (2.2)	1.01 (0.74–1.37)	0.950
**Glucosamine**								
All-cause mortality	218 (15.9)	1,447 (23.6)	0.69 (0.60–0.80)	** < 0.001**	238 (14.0)	801 (14.0)	1.00 (0.86–1.16)	0.986
Cancer mortality	173 (12.6)	1,136 (18.6)	0.71 (0.61–0.84)	** < 0.001**	197 (11.6)	698 (12.2)	0.96 (0.82–1.13)	0.620
CVD mortality	49 (3.6)	367 (6.0)	0.71 (0.52–0.97)	**0.030**	39 (2.3)	135 (2.4)	0.99 (0.69–1.42)	0.970

^a^All models adjusted for age, sex, ethnicities, socio-economic (Townsend deprivation index score and education level), alcohol status, time since cancer diagnosis, CVD diagnosis prior to assessment, vitamin or mineral supplement use, Charlson comorbidity index, cancer prognosis, oily fish consumption (only for fish oil). Class 1, characterized by a Life Essential score at or below the mean. Class 2, characterized by scores above the mean

Significant interactions on an additive scale were found between regular fish oil use and LE8 scores on all-cause and cancer mortality (RERI = 0.33–0.40) (Supplementary Fig. 1). For glucosamine, the interactions between regular use and LE8 scores on all-cause, cancer and CVD-related mortality were all found to be significant on an additive scale (RERI = 0.91–1.23), whereas the interactions on all-cause and cancer mortality were also significant on a multiplicative scale (both *P* for interaction < 0.01). In the joint analysis (Supplementary Fig. 1), compared to the reference group (i.e., non-fish oil/non-glucosamine users with lower LE8 scores), all groups exhibit significantly lower risks of all-cause, cancer and CVD-related mortality (all *P* for interaction < 0.05), except for no significant differences in CVD-related mortality among regular fish oil users with lower LE8 scores. The lowest risk of mortality was observed among participants who were regular users of fish oil or glucosamine and had a higher LE8 score.

### Associations of fish oil and glucosamine use with mortality in participants with *cancer* with different prognoses

The associations between the use of fish oil or glucosamine and mortality were only found to be significant in participants with poor cancer prognoses (Table 5). In this subgroup of participants, the use of fish oil was associated with reduced risks of all-cause mortality (aHR = 0.75, 95% CI = 0.66–0.86, *p* < 0.001) and cancer mortality (aHR = 0.77, 95% CI = 0.67–0.89, *p* = 0.001), but not CVD-related mortality. Similarly, glucosamine use was associated with reduced risks of all-cause mortality (aHR = 0.74, 95% CI = 0.62–0.87, *p* < 0.001) and cancer mortality (aHR = 0.71, 95% CI = 0.59–0.86, *p* < 0.001). However, no significant associations were observed between the use of fish oil or glucosamine use and the risk of mortality in the group of participants with good cancer prognoses. The results regarding the combined use of fish oil and glucosamine were consistent with those of the individual supplements (Supplementary Table 5).

**Table 5:** Associations of fish oil and glucosamine use with risks of overall, cancer and CVD mortality stratified by cancer prognoses

	**Death among users**	**Death among non-users**	**Cancer with good prognosis (*n* = 10,229)**		**Death among users**	**Death among non-users**	**Cancer with poor prognosis (*n* = 4,691)**	
	N (%)	N (%)	Hazard ratio (95% CI)	*P*			Hazard ratio (95% CI)	*P*
**Fish oil**								
All-cause mortality	534 (14.7)	855 (13.4)	1.02 (0.91–1.14)	0.758	332 (22.9)	953 (29.4)	0.75 (0.66–0.86)	** < 0.001**
Cancer mortality	425 (11.7)	725 (11.0)	1.01 (0.89–1.15)	0.870	271 (18.7)	783 (24.2)	0.77 (0.67–0.89)	**0.001**
CVD mortality	116 (3.2)	166 (2.5)	1.16 (0.90–1.49)	0.240	87 (6.0)	221 (6.8)	0.89 (0.68–1.17)	0.410
**Glucosamine**								
All-cause mortality	291 (12.8)	1,128 (14.2)	0.89 (0.78–1.02)	0.094	165 (21.0)	1120 (38.7)	0.74 (0.62–0.87)	** < 0.001**
Cancer mortality	240 (10.5)	910 (11.5)	0.91 (0.79–1.05)	0.210	130 (16.6)	924 (23.6)	0.71 (0.59–0.86)	** < 0.001**
CVD mortality	52 (2.3)	230 (2.9)	0.88 (0.64–1.19)	0.400	36 (4.6)	272 (7.0)	0.74 (0.51–1.06)	0.100

^a^Categorized into 2 groups based on average prognosis and statistics in the UK Biobank. Types of cancer with better average prognosis are classified into one group (Malignant neoplasms of male or female genital organs, malignant melanoma of skin, malignant neoplasms of thyroid and other endocrine glands, malignant neoplasms of breast) and other types with poorer average prognosis are classified into another group. All models adjusted for age, sex, ethnicities, socio-economic (Townsend deprivation index score and education level), Life 8 essential scores, alcohol status, time since cancer diagnosis, CVD diagnosis prior to assessment, vitamin or mineral supplement use, Charlson comorbidity index, oily fish consumption (only for fish oil)

Significant interactions on both additive (RERI = 1.11–1.12) and multiplicative scales (both *P* for interaction < 0.005) were found between regular fish oil use and cancer prognosis on all-cause and cancer mortality (Supplementary Fig. 1). Similarly for glucosamine, the interactions between regular use and cancer prognosis on all-cause and cancer mortality were all found to be significant on additive (RERI = 0.99–1.15) and multiplicative scales (both *P* for interaction < 0.05). In the joint analysis (Supplementary Fig. 1), compared to the reference group (i.e., non-fish oil/non-glucosamine users with poor cancer prognosis), most of the groups exhibit significantly lower risks of all-cause, cancer and CVD-related mortality (all *P* for interaction < 0.05), except for no significant differences in CVD-related mortality among regular fish oil or glucosamine users who had cancers of poor prognosis. The lowest risk of mortality was observed among participants who were regular users of fish oil or glucosamine and had cancers with good prognosis.

## Discussion

This is the first study to examine the impact of Life Essential 8 scores and cancer prognosis on the associations between the use of fish oil and glucosamine and the risk of mortality in a cancer patient population. The findings indicate that both glucosamine and fish oil supplementation were associated with decreased risks of all-cause and cancer mortality. While previous research has demonstrated the potential benefits of dietary supplements on mortality in cancer survivors [8, 65], no studies have explored the effects of fish oil or glucosamine in different subgroups of patients based on their lifestyle and clinical characteristics. Adding to the existing evidence, this study offers novel findings that these associations were observed in specific subgroups of patients, mainly patients with low LE8 scores and those with poor cancer prognoses. This suggests that the benefits of dietary supplementation on mortality may vary among patients with cancer, implying that a one-size-fits-all approach may not be ideal when considering supplementation. By identifying patient characteristics that influence the benefits of supplementation against mortality using real-world data, personalized integrative cancer counselling can be developed to support patients with cancer. The real-world findings can also inform future confirmatory clinical trials that assess the benefits of supplementation on the basis of the unique needs and characteristics of patients [35].

Consistent with studies conducted in the general population [18–21], this study found lower risks of all-cause and cancer mortality in patients with cancer who used fish oil or glucosamine supplements than in those who did not. However, the significant associations between the use of these supplements and reduced mortality risks were only observed in patients with LE8 scores lower than the mean score, which refer to patients who have comparatively poorer lifestyle and cardiovascular health. Studies conducted on the general population have found that the strength of association between supplement use and mortality risk can be affected by various lifestyle or health factors, such as dietary quality, smoking, cholesterol levels, or the presence of hypertension and diabetes [18, 20, 21, 66]. However, it is still unclear whether lifestyle or the varying levels of cardiovascular health, could impact the associations between supplement use and mortality risk. These individual factors also may not fully represent the overall health status. Moreover, the patterns of lifestyle behaviours observed in patients with cancer differ from those seen in the non-cancer population [67, 68]. In contrast to previous studies, our study used a combination of metrics of both lifestyle and cardiovascular health factors, thereby defining ideal cardiovascular health as having all metrics at optimal levels rather than focusing on a single factor [33]. This approach aligns better with real-world scenarios, as patients with a healthier lifestyle usually adhere to more than one recommendation [69].

Previous studies have reported conflicting results regarding the associations between the use of glucosamine and fish oil (or omega-3 fatty acid) supplements and CVD-related mortality [18, 19, 21, 39]. In our study, neither glucosamine nor fish oil was found to be significantly associated with a decreased risk of CVD-related mortality in the fully adjusted model. However, among patients with LE8 scores lower than the mean score, glucosamine use was found to be associated with a significantly reduced risk of CVD-related mortality. Glucosamine may potentially reduce systematic inflammation [26]. Previous studies have shown that higher inflammation, such as taking pro-inflammatory diet, may lead to increased all-cause and cancer mortality [23, 24], as well as poorer prognosis in older cancer patients [70] although the exact pathways have not been fully elucidated. Patients with unhealthy lifestyles tend to have higher levels of inflammation than those with healthy lifestyles [71, 72]. A low LE8 score also reflects poor cardiovascular health, which was also found to be associated with high levels of inflammation [73]. Studies have reported that glucosamine use is associated with a decreased risk of CVDs [36, 74], particularly among patients with unhealthy lifestyles [74]. The findings from the present study imply that glucosamine provides cardiovascular benefits for patients with low LE8 scores. Nevertheless, these benefits appear to be negligible among patients who already have better-than-average cardiovascular health status.

We also observed that the association between the use of fish oil or glucosamine supplements and mortality was significant in the group of patients with poor cancer prognoses, but not in the group with good cancer prognoses. To the best of our knowledge, no study has investigated the influence of cancer prognosis on the effects of supplements, particularly fish oil and glucosamine, on the survival of patients with cancer. A meta-analysis of vitamin D supplementation suggested that while it did not reduce overall cancer mortality, it was associated with a decreased risk of mortality due to lung cancer, which typically has a poor prognosis [75]. Our findings suggest that glucosamine and fish oil supplementation are associated with a certain extent of mortality risk reduction, particularly when the cancer prognosis at baseline is poor, but the effect may be negligible in cases with a more favorable prognosis. The results of this study demonstrate the potential for personalized interventions involving dietary supplements. However, at present, our understanding of the potential benefits or harms of individual dietary supplements, particularly in relation to subgroups of patients with specific characteristics, remains limited. In the case of fish oil and glucosamine, it may be prudent to allow patients who have low LE8 scores or poor cancer prognoses to consider supplementation due to the relatively good safety profiles of these supplements. Nevertheless, it is crucial to emphasize that this should be encouraged in conjunction with other interventions, such as exercise, nutrition counseling, and sleep education. As indicated by our joint effect analysis, the reduction in mortality risk was more pronounced in the group with a higher LE8 score (irrespective of supplement use status) than fish oil or glucosamine users with lower LE8 scores.

Despite using a large sample and a prospective observational design with well-characterized mortality outcomes, this study has some limitations. First, the use of dietary supplements after cancer diagnosis was self-reported, potentially leading to recall inaccuracies. Some information, including cancer treatment and stage, was not available. However, studies have shown that the effect of staging at cancer diagnosis on overall survival largely disappeared after having survived for 5 to 10 years [76]. The dosage and duration of supplements were also not available in the UK Biobank. Future studies should include such clinical and treatment data and relevant questions to obtain a more complete picture of supplement use and cancer status by patients with cancer. Furthermore, the UK Biobank study is known to have a low participation rate and a selection bias toward healthy volunteers with relatively low mortality rates and healthy lifestyles [77]. However, many studies have shown that this cohort may still provide valid inferences of risk factors and exposure–disease associations that are generalizable [77, 78].

## Conclusions

This large prospective cohort study showed that the use of fish oil and glucosamine was associated with decreased all-cause mortality and cancer mortality among patients with cancer. Specifically, the association was significant in patients with LE8 scores lower than the mean score (poorer lifestyle and cardiovascular health) and in patients with poorer cancer prognoses. Additionally, glucosamine supplementation was found to be associated with a lower risk of CVD-related mortality only in patients with lower LE8 scores. These findings highlight the potential differential impact of lifestyle factors, cardiovascular health, and cancer prognosis on the associations between supplement use and mortality risk in patients with cancer. Future research should take these factors into account when investigating the effects of supplements on health outcomes using real-world data. Such information can help clinicians to guide patients in making informed decisions about dietary supplementation and enable the provision of personalized integrative cancer care.

## Supplementary Information

Supplementary Material 1.

## Data Availability

The datasets analyzed during the current study are available in the UK Biobank Repository. This research has been conducted using the UK Biobank Resource under Application Number 74158.
