# Supplementary material for: Association between fish oil and glucosamine use and mortality in patients diagnosed with cancer: the role of the Life Essential 8 score and cancer prognosis
Source: Nutr J. 2024 Oct 17;23:124. doi: 10.1186/s12937-024-01032-1 (PMC11484474; doi:10.1186/s12937-024-01032-1)
Supplement: Supplementary file 1 — Supplementary Material 1. [file 12937_2024_1032_MOESM1_ESM.docx]

**Supplementary Table 1: Details on the data collection and ascertainment of dietary supplement use and other selected confounders**

| **Variable** | **Description** | **Categories** |
| --- | --- | --- |
| **Use of vitamin or mineral supplements** | In the questions from the touchscreen questionnaire related to the use of supplements, participants were asked "Do you regularly take any of the following?". They were provided lists of supplements, one included vitamin supplements, another included mineral, fish oil, and glucosamine. They could select more than one answer or “None of the above” if they did not regularly take any of the listed supplements. | Vitamin A, B, C, D, E, multivitamins ± minerals, folic acid, calcium, zinc, selenium, iron |
| **Townsend Deprivation Index** | A composite indicator of participants’ socio-economic status based on home ownership, car ownership, employment status, and household crowdedness.^1^ |  |
| **Education** |  | Degree (college/university degree) or below degree |
| **BMI** | BMI was calculated by dividing participant’s weights in kg (using the Tanita BC-418 MA body composition analyzer), by the square of their standing heights in meters, measured with a Seca 202 device. |  |
| **Smoking and alcohol consumption** |  | Self-reported as never, former, or current at the point of recruitment |
| **Comorbidities** | The UK Biobank is linked to health-related records including hospital inpatient data, coded primary care data, and death registry data. We did not consider records consisting only of self-reported data^2^.  Charlson Comorbidity Index was used to assess the comorbidities of participants prior to cancer diagnosis. The algorithm by Quan et al^3^ was employed to calculate the Charlson Index as the health records in the UK Biobank were defined by the ICD-9 and ICD-10 codes. | We used 2 as a cut-off point for Charlson Comorbidity Index in effect modification analyses as all participants included had history of cancer which corresponded to a score of 2. |
| **Cancer prognosis** | Cancer diagnoses were coded according to the ICD-9 and ICD-10 codes. We included only malignant neoplasms (ICD-9: 140–208; ICD-10: C00–C97), except non-melanoma skin cancer (ICD-9: 173; ICD-10: C44). | Categorized into 2 groups based on average prognosis^4,5^ and statistics in the UK Biobank. Types of cancer with better average prognosis (mortality rate < 25% and higher survival rate) are classified into one group (Malignant neoplasms of male or female genital organs, malignant melanoma of skin, malignant neoplasms of thyroid and other endocrine glands, malignant neoplasms of breast) and other types with poorer average prognosis (mortality rate > 25% and lower survival rate) are classified into another group. |

^a^ Sociodemographic and lifestyle characteristics were defined at baseline; however, if the participants had missing data for any of the baseline characteristics at recruitment, the earliest responses at follow-up visits were used.

References:

1. Jarman B, Townsend P, Carstairs V. Deprivation indices. *BMJ*. 1991;303(6801):523.

2. UK Biobank. First occurrence of health outcomes defined by 3-character ICD10 code. 2019. Accessed Mar 1, 2024. https://biobank.ndph.ox.ac.uk/showcase/showcase/docs/first_occurrences_outcomes.pdf

3. Quan H, Sundararajan V, Halfon P, et al. Coding algorithms for defining comorbidities in ICD-9-CM and ICD-10 administrative data. *Medical care*. 2005:1130-9.

4. Cancer Research UK. Cancer statistics for the UK. 2022. Accessed Mar 1, 2024. https://www.cancerresearchuk.org/health-professional/cancer-statistics/statistics-by-cancer-type

5. National Health Service UK. Cancer Survival in England, cancers diagnosed 2015 to 2019, followed up to 2020. 2022. Accessed Mar 1, 2024. https://digital.nhs.uk/data-and-information/publications/statistical/cancer-survival-in-england/cancers-diagnosed-2015-to-2019-followed-up-to-2020/survival-by-cancer-group

# **Supplementary Table 2. Life's Essential 8 score metrics from the American Heart Association adapted for the UK Biobank**

| **Health Metric** | **Information** | **Scoring** | |
| --- | --- | --- | --- |
|  |  | **Points** | **Status** |
| Smoking | Smoking was classified using self-reported information. | 100  50  0 | Never  Previous  Current |
| Body mass index | Body weight (kg) divided by height squared (m^2^) | 100  70  30  15  0 | <25 kg/m^2^  25.0 to 29.9 kg/m^2^  30.0 to 34.9 kg/m^2^  35.0 to 39.9 kg/m^2^  ≥40.0 kg/m^2^ |
| Physical activity | Self-reported physical activity converted to MET/week | 100  90  80  60  40  20  0 | ≥600 MET/min/week  ≥480 to <600 MET/min/week  ≥360 to <480 MET/min/week  ≥240 to <360 MET/min/week  ≥120 to <240 MET/min/week  ≥4 to <120 MET/min/week  0 MET/min/week |
| Healthy diet score | Diet score created for the UK Biobank data (9 points) and split into quartiles | 100  50  25  0 | Highest quartile (score ≥7)  3^rd^ quartile (score between 5 and 6)  2^nd^ quartile (score =4)  Lowest quartile (score <3) |
| Non-HDL cholesterol | Plasma total and HDL cholesterol to estimate non-HDL cholesterol* | 100  60  40  20  0 | <130 mg/dl  ≥130 to 189 mg/dl  ≥160-189 mg/dl  ≥190 to 219 mg/dl  ≥220 mg/dl |
| Blood pressure | Appropriately measured systolic and diastolic blood pressure* | 100  75  50  25  0 | <120 & <80 mmHg  ≥120 to 129 & <80 mmHg  ≥130 to 139 or ≥80 to 89 mmHg  ≥140 to 159 or ≥90 to 99 mmHg  ≥160 or ≥100 mmHg |
| HbA1c | Casual HbA1c | 100  60  40  30  20  10  0 | <5.7 %  ≥5.7 to 6.4 %  Diabetes and HbA1c <7.0%  Diabetes and HbA1c ≥7.0 to 7.9%  Diabetes and HbA1c ≥8.0 to 8.9%  Diabetes and HbA1c ≥9.0 to 9.9%  Diabetes and HbA1c ≥10.0% |
| Sleep | Self-reported average hours of sleep per night | 100  90  70  40  20  0 | ≥7 to <9 h/day  ≥9 to <10 h/day  ≥6 to <7 h/day  ≥5 to <6 or ≥10 h/day  ≥4 to <5 h/day  <4 h/day |

*20 points were subtracted when people in any category were using medication/treatment.

Reference:

1. Petermann-Rocha F, Deo S, Celis-Morales C, Ho FK, Bahuguna P, McAllister D, et al. An opportunity for prevention: associations between the Life's Essential 8 score and cardiovascular incidence using prospective data from UK Biobank*. Current problems in cardiology*. 2023;48(4):101540.

2. Petermann-Rocha F, Ho FK, Foster H, Boopor J, Parra-Soto S, Gray SR, et al., editors. Nonlinear associations between cumulative dietary risk factors and cardiovascular diseases, cancer, and all-cause mortality: a prospective cohort study from UK Biobank. *Mayo Clinic Proceedings*; 2021;96(9):2418-2431.

**Individual food items and their categorisation**

| **Variable** | **Categories reported from the touch-screen questionnaire** | **Binary Variables:** |
| --- | --- | --- |
| Fruit & vegetables (regrouped from fruit, dried fruit & Vegetable) | Serving/day | ≥5 serving/day (Ref.)  <5 serving/day |
| Total fish intake (regrouped from Both total non-oily fish and oily fish) | Never  Less than once a week  Once a week  2-4 times a week  5-6 times a week  Once or more daily | ≥2 times a week (at least once a week of each category)  (Ref.)  < once a week of each one |
| Processed meat intake | Never  Less than once a week  Once a week  2-4 times a week  5-6 times a week  Once or more daily | ≤Once a week  (Ref.)  > Once a week |
| Red meat (regrouped from beef, pork and lamb) | Never  Less than once a week  Once a week  2-4 times a week  5-6 times a week  Once or more daily | >Once a week (Ref.)  ≤Once a week |
| Milk type used | Full cream  Semi-skimmed  Skimmed  Soya  another type of milk  Never rarely have milk | Semi-skimmed/skimmed (Ref.)  Full cream/ another type of milk/ never rarely have milk |
| Spread type | Never/rarely  Butter  Other type/ margarine  Flora pro-active/benecol | Never/rarely (Ref.)  Another selection |
| Cereal intake | Bowls/week | >5 bowls (Ref)  ≤5 bowls |
| Salt added to food | Never/rarely  Sometimes  Usually  Always | Never/rarely (Ref.)  Another selection |
| Water intake | Glasses/day | ≥6 glasses (Ref.)  <6 glasses |

**Supplementary Table 3**. **Baseline characteristics of included participants (N=14,920)**

|  | Overall  (n=14,920) | | % |
| --- | --- | --- | --- |
| **Sociodemographics** |  | |  |
| **Sex** |  | |  |
| Male | 5,940 | | 39.8 |
| Female | 8,980 | | 60.2 |
| **Age attending assessment centres (Mean ± SD)** | 59.9 | | ±7.1 |
| **Townsend deprivation index (Mean ± SD)** | -1.6 | | ±2.9 |
| **Ethnic background** |  | |  |
| White | 14,557 | | 97.6 |
| Others | 363 | | 2.4 |
| **Education** |  | |  |
| College or university degree | 4,839 | | 32.4 |
| Below degree | 10,081 | | 67.6 |
| Life Essential 8 scores |  | |  |
| Total score (Mean ± SD) | 64.0 | | ±12.1 |
| BMI score (Mean ± SD) | 70.1 | | ±28.0 |
| Nicotine exposure score (Mean ± SD) | 71.4 | | ±32.1 |
| Physical activity score (Mean ± SD) | 74.6 | | ±37.7 |
| Sleep health score (Mean ± SD) | 89.0 | | ±19.1 |
| Diet score (Mean ± SD) | 33.9 | | ±28.5 |
| Blood lipids score (Mean ± SD) | 47.3 | | ±29.0 |
| Blood glucose score (Mean ± SD) | 89.6 | | ±20.3 |
| Blood pressure score (Mean ± SD) | 36.4 | | ±27.0 |
| Lifestyle |  |  | |
| BMI (Mean ± SD) | 27.2 | | ±4.6 |
| Smoking status |  | |  |
| Never | 7,647 | | 51.3 |
| Former | 6,021 | | 40.4 |
| Current | 1,252 | | 8.4 |
| Alcohol consumption |  | |  |
| Never | 579 | | 3.9 |
| Former | 647 | | 4.3 |
| Current | 13,694 | | 91.8 |
| Vitamin or mineral supplement use | 6,373 | | 42.7 |
| Clinical |  |  | |
| Prior CVD diseases | 1,126 | | 7.5 |
| Cancer diagnoses (Top 5) |  | |  |
| Breast | 4,889 | | 32.8 |
| Genitourinary | 3,073 | | 20.6 |
| Digestive organs/Gastrointestinal | 1,704 | | 11.4 |
| Melanoma | 1,302 | | 8.7 |
| Hematological | 1,251 | | 8.4 |
| Class of cancer diagnoses |  | |  |
| C00-C14 lip, oral cavity and pharynx | 308 | | 2.1 |
| C15-C26 digestive organs | 1,704 | | 11.4 |
| C30-C39 respiratory and intrathoracic organs | 319 | | 2.1 |
| C40-C41 bone and articular cartilage | 63 | | 0.4 |
| C43 Malignant melanoma of skin | 1,302 | | 8.7 |
| C45-C49 mesothelial and soft tissue | 149 | | 1.0 |
| C50 breast | 4,889 | | 32.8 |
| C51-C58 female genital organs | 1,377 | | 9.2 |
| C60-C63 male genital organs | 2,415 | | 16.2 |
| C64-C68 urinary tract | 661 | | 4.4 |
| C69-C72 eye, brain and other parts of CNS | 162 | | 1.1 |
| C73-C75 thyroid and other endocrine glands | 257 | | 1.7 |
| C76-C80 ill-defined, secondary and unspecified sites | 133 | | 0.9 |
| C81-C96 primary, of lymphoid, haematopoietic and related tissue | 1,251 | | 8.4 |
| Age at first cancer diagnosis (Mean ± SD) | 52.4 | | ±10.3 |
| Year since cancer diagnosis (Median [IQR)) | 6.0 | | 2.0-11.0 |

**Supplementary Table 4. Sensitivity analyses on the association of fish oil and glucosamine use with risk of overall, cancer and CVD mortality**

|  |  | | Model 1 (Crude) | | | Model 2 ^a^ | | Model 3 ^a^ |  |
| --- | --- | --- | --- | --- | --- | --- | --- | --- | --- |
|  | Death among users | Death among non-users | | Hazard ratio (95% CI) | *P* | Hazard ratio (95% CI) | *P* | Hazard ratio (95% CI) | *P* |
| *Without considering competing risks (n=14,920)* |  |  | |  |  |  |  |  |  |
| Fish oil |  |  | |  |  |  |  |  |  |
| Cancer mortality | 696 (13.7) | 1508 (15.3) | | 0.88 (0.80-0.96) | **0.004** | 0.82 (0.75-0.90) | **<0.001** | 0.89 (0.81-0.98) | **0.018** |
| CVD mortality | 203 (4.0) | 387 (3.9) | | 0.99 (0.84-1.17) | 0.910 | 0.87 (0.73-1.03) | 0.116 | 0.99 (0.82-1.19) | 0.917 |
| Glucosamine |  |  | |  |  |  |  |  |  |
| Cancer mortality | 370 (12.1) | 1834 (15.5) | | 0.76 (0.68-0.85) | **<0.001** | 0.75 (0.67-0.84) | **<0.001** | 0.82 (0.73-0.92) | **<0.001** |
| CVD mortality | 88 (2.9) | 502 (4.2) | | 0.66 (0.53-0.83) | **<0.001** | 0.63 (0.51-0.81) | **<0.001** | 0.80 (0.63-1.01) | 0.056 |
| *Remove death within 2 years (n=14,449)* |  |  | |  |  |  |  |  |  |
| Fish oil |  |  | |  |  |  |  |  |  |
| All-cause mortality | 728 (14.7) | 1507 (15.9) | | 0.91 (0.84-1.00) | **0.044** | 0.84 (0.76-0.91) | **<0.001** | 0.87 (0.79-0.96) | **0.002** |
| Cancer mortality | 571 (11.5) | 1205 (12.7) | | 0.90 (0.81-0.99) | **0.035** | 0.83 (0.76-0.92) | **<0.001** | 0.88 (0.79-0.98) | **0.007** |
| CVD mortality | 184 (3.7) | 344 (3.6) | | 1.02 (0.85-1.22) | 0.830 | 0.91 (0.76-1.08) | 0.280 | 0.99 (0.82-1.20) | 0.960 |
| Glucosamine |  |  | |  |  |  |  |  |  |
| All-cause mortality | 395 (13.1) | 1840 (16.1) | | 0.81 (0.72-0.90) | **<0.001** | 0.78 (0.70-0.87) | **<0.001** | 0.83 (0.75-0.93) | **0.002** |
| Cancer mortality | 314 (10.4) | 1462 (12.8) | | 0.81 (0.72-0.92) | **0.001** | 0.79 (0.69-0.89) | **<0.001** | 0.83 (0.74-0.94) | **0.004** |
| CVD mortality | 83 (2.8) | 445 (3.9) | | 0.71 (0.56-0.90) | **0.004** | 0.69 (0.55-0.87) | **0.002** | 0.82 (0.64-1.04) | 0.110 |

^a^ Model 2: adjusted for age and sex; Model 3: adjusted for age, sex, ethnicities, socio-economic (Townsend deprivation index score and education level), Life 8 essential scores, alcohol status, time since cancer diagnosis, CVD diagnosis prior to assessment, vitamin or mineral supplement use, Charlson comorbidity index, cancer prognosis, oily fish consumption (only for fish oil)

**Supplementary Table 5. Association of the combined use of fish oil and glucosamine use with risk of overall, cancer and CVD mortality, and analyses stratified by Life 8 Essential Score class and cancer prognoses**

| Main analysis (n=10,743) | Death among users | Death among non-users | Model 1 (Crude) |  | Model 2 ^a^ |  | Model 3 ^a^ |  |
| --- | --- | --- | --- | --- | --- | --- | --- | --- |
|  | N (%) | N (%) | Hazard ratio (95% CI) | *P* | Hazard ratio (95% CI) | *P* | Hazard ratio (95% CI) | *P* |
| All-cause mortality | 299 (15.0) | 1681 (19.2) | 0.76 (0.67-0.86) | **<0.001** | 0.70 (0.62-0.80) | **<0.001** | 0.79 (0.69-0.90) | **<0.001** |
| Cancer mortality | 249 (12.5) | 1387 (15.8) | 0.77 (0.68-0.88) | **<0.001** | 0.73 (0.64-0.83) | **<0.001** | 0.81 (0.70-0.94) | **0.004** |
| CVD mortality | 56 (2.8) | 355 (4.1) | 0.69 (0.52-0.92) | **0.010** | 0.62 (0.47-0.82) | **0.001** | 0.80 (0.59-1.09) | 0.160 |

^a^ Model 2: adjusted for age and sex; Model 3: adjusted for age, sex, ethnicities, socio-economic (Townsend deprivation index score and education level), Life 8 essential scores, alcohol status, time since cancer diagnosis, CVD diagnosis prior to assessment, vitamin or mineral supplement use, Charlson comorbidity index, cancer prognosis, oily fish consumption

|  | Death among users | Death among non-users | Class 1 (Mean score or below)  (n=5,509) |  | Death among users | Death among non-users | Class 2 (Above mean score)  (n=5,234) |  |
| --- | --- | --- | --- | --- | --- | --- | --- | --- |
|  | N (%) | N (%) | Hazard ratio (95% CI) | *P* | N (%) | N (%) | Hazard ratio (95% CI) | *P* |
| All-cause mortality | 147 (16.4) | 1097 (23.8) | 0.69 (0.58-0.83) | **<0.001** | 152 (13.9) | 584 (14.1) | 0.89 (0.73-1.07) | 0.214 |
| Cancer mortality | 121 (13.5) | 871 (18.9) | 0.76 (0.62-0.93) | **0.007** | 128 (11.7) | 516 (12.5) | 0.86 (0.70-1.06) | 0.150 |
| CVD mortality | 32 (3.6) | 265 (5.7) | 0.71 (0.48-1.07) | 0.100 | 24 (2.2) | 90 (2.2) | 0.97 (0.62-1.53) | 0.900 |

^a^ All models adjusted for age, sex, ethnicities, socio-economic (Townsend deprivation index score and education level), alcohol status, time since cancer diagnosis, CVD diagnosis prior to assessment, vitamin or mineral supplement use, Charlson comorbidity index, cancer prognosis, oily fish consumption. Class 1, characterized by a Life Essential score at or below the mean, indicates poorer cardiovascular health. Class 2, characterized by scores above the mean, indicates better cardiovascular health.

|  | Death among users | Death among non-users | Cancer with good prognosis (n=7,255) |  | Death among users | Death among non-users | Cancer with poor prognosis (n=3,488) |  |
| --- | --- | --- | --- | --- | --- | --- | --- | --- |
|  | N (%) | N (%) | Hazard ratio (95% CI) | *P* | N (%) | N (%) | Hazard ratio (95% CI) | *P* |
| All-cause mortality | 198 (13.4) | 792 (13.7) | 0.92 (0.78-1.09) | 0.360 | 101 (19.6) | 889 (29.9) | 0.62 (0.50-0.77) | **<0.001** |
| Cancer mortality | 167 (11.3) | 652 (11.3) | 0.96 (0.80-1.16) | 0.690 | 82 (15.9) | 735 (24.7) | 0.62 (0.49-0.79) | **<0.001** |
| CVD mortality | 35 (2.4) | 149 (2.6) | 0.98 (0.67-1.43) | 0.920 | 21 (4.1) | 206 (6.9) | 0.63 (0.39-1.03) | 0.067 |

^a^ Categorized into 2 groups based on average prognosis and statistics in the UK Biobank^30,31^. Types of cancer with better average prognosis are classified into one group (Malignant neoplasms of male or female genital organs, malignant melanoma of skin, malignant neoplasms of thyroid and other endocrine glands, malignant neoplasms of breast) and other types with poorer average prognosis are classified into another group. All models adjusted for age, sex, ethnicities, socio-economic (Townsend deprivation index score and education level), Life 8 essential scores, alcohol status, time since cancer diagnosis, CVD diagnosis prior to assessment, vitamin or mineral supplement use, Charlson comorbidity index, oily fish consumption

**Supplementary Figure 1. Joint analysis of the effects of regular use of fish oil or glucosamine supplements and LE8 score or cancer prognosis on all-cause mortality, cancer and CVD mortality**

**
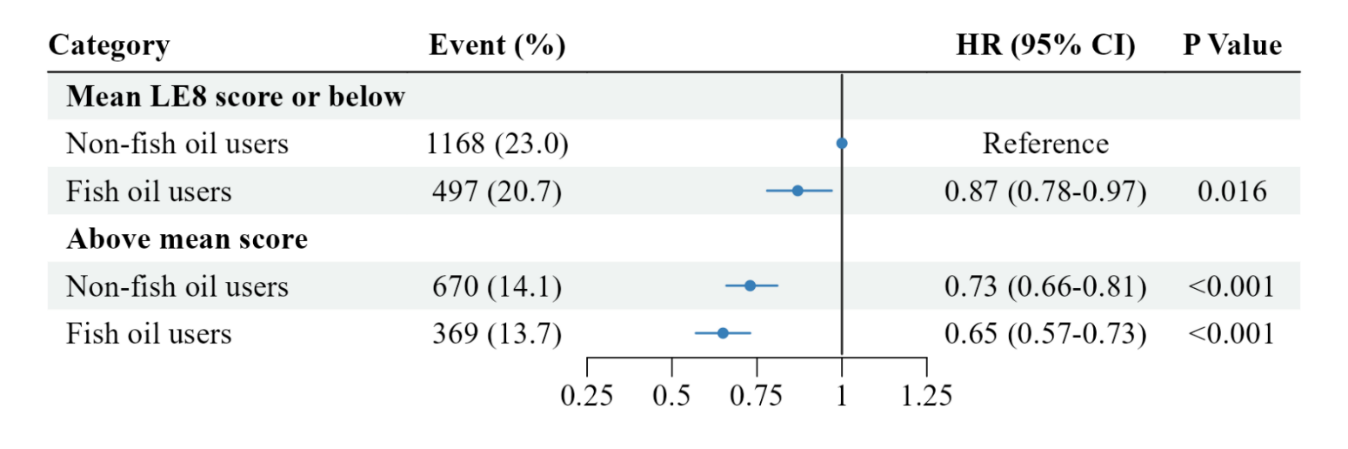
**

**Joint analysis of the effects of regular use of fish oil supplements and LE8 score on all-cause mortality** Participants were classified into four groups based on their regular fish oil use status (non-users or users) and LE8 score (mean LE8 score of the cohort=64.0 as the cut-off).

Measure of interaction on additive scale (RERI, 95% CI): 0.40 (0.12-0.69).

Measure of interaction on multiplicative scale (95% CI): 1.01 (0.86-1.19), *P* for interaction=0.904.


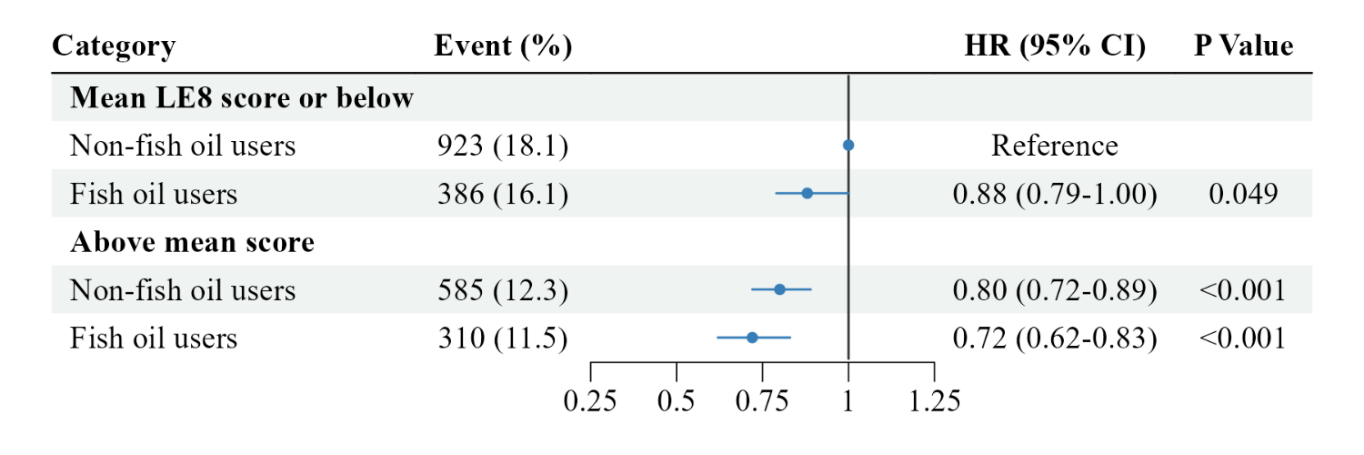


**Joint analysis of the effects of regular use of fish oil supplements and LE8 score on cancer mortality** Participants were classified into four groups based on their regular fish oil use status (non-users or users) and LE8 score (mean LE8 score of the cohort=64.0 as the cut-off).

Measure of interaction on additive scale (RERI, 95% CI): 0.33 (0.01-0.65).

Measure of interaction on multiplicative scale (95% CI): 1.00 (0.83-1.20), *P* for interaction=0.998.


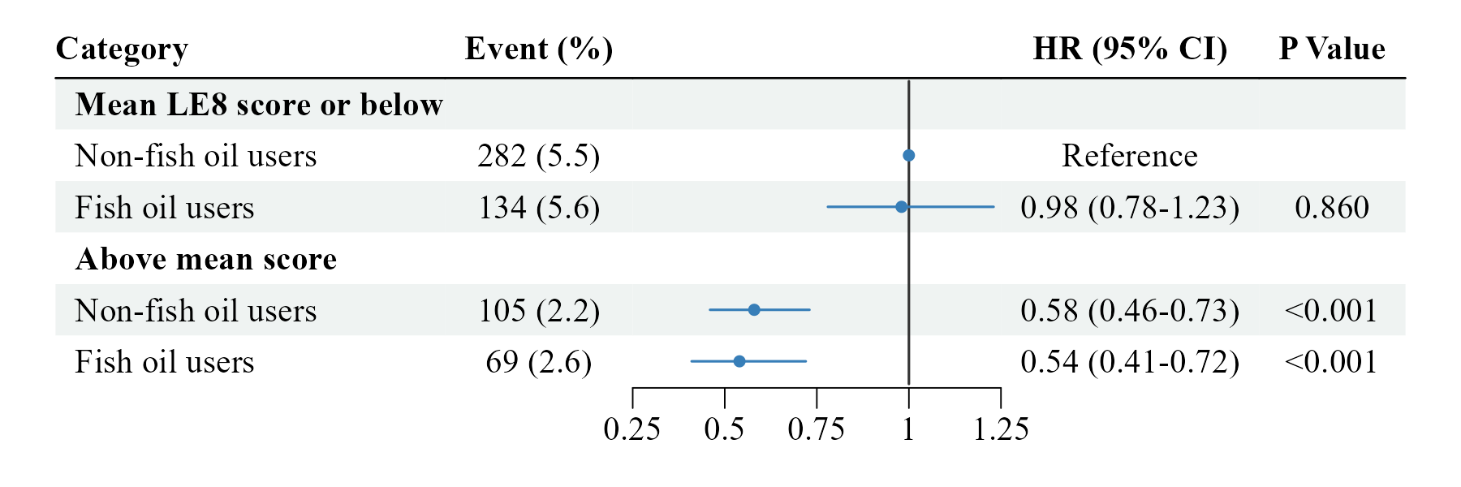


**Joint analysis of the effects of regular use of fish oil supplements and LE8 score on CVD-related mortality** Participants were classified into four groups based on their regular fish oil use status (non-users or users) and LE8 score (mean LE8 score of the cohort=64.0 as the cut-off).

Measure of interaction on additive scale (RERI, 95% CI): 0.51 (-0.09-1.10).

Measure of interaction on multiplicative scale (95% CI): 0.96 (0.77-1.19), *P* for interaction=0.714.


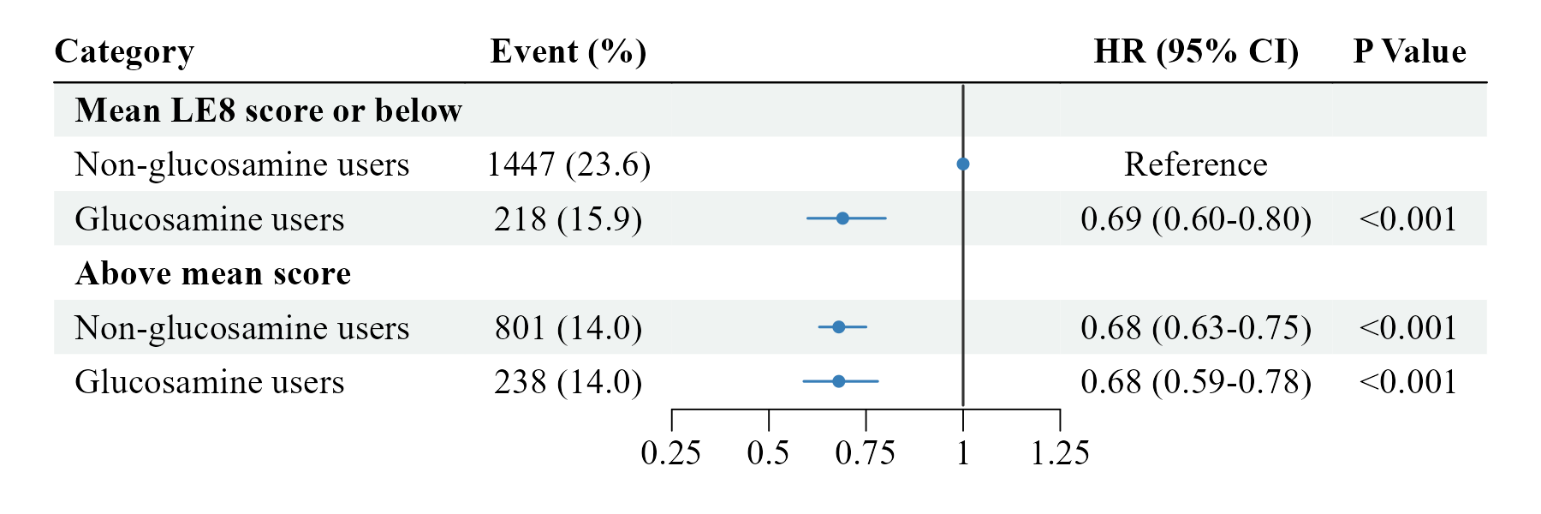


**Joint analysis of the effects of regular use of glucosamine supplements and LE8 score on all-cause mortality** Participants were classified into four groups based on their regular glucosamine use status (non-users or users) and LE8 score (mean LE8 score of the cohort=64.0 as the cut-off).

Measure of interaction on additive scale (RERI, 95% CI): 1.05 (0.66-1.45).

Measure of interaction on multiplicative scale (95% CI): 1.43 (1.17-1.75), *P* for interaction<0.001.


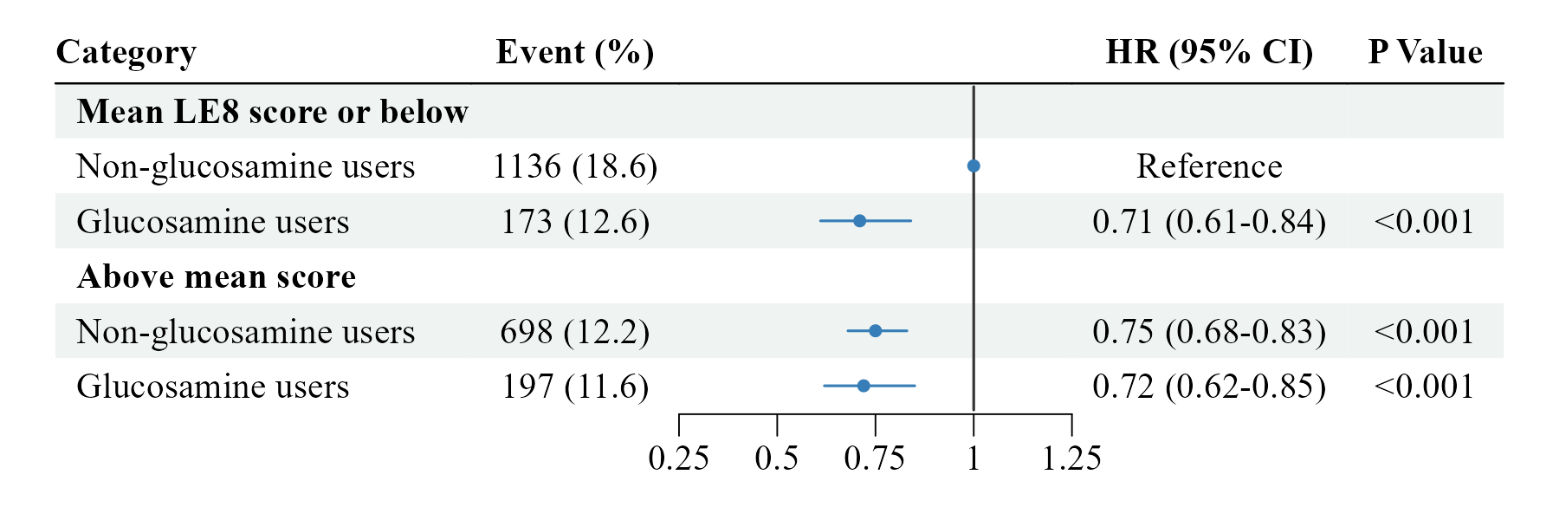


**Joint analysis of the effects of regular use of glucosamine supplements and LE8 score on cancer mortality** Participants were classified into four groups based on their regular glucosamine use status (non-users or users) and LE8 score (mean LE8 score of the cohort=64.0 as the cut-off).

Measure of interaction on additive scale (RERI, 95% CI): 0.91 (0.49-1.34).

Measure of interaction on multiplicative scale (95% CI): 1.36 (1.08-1.70), *P* for interaction=0.008.


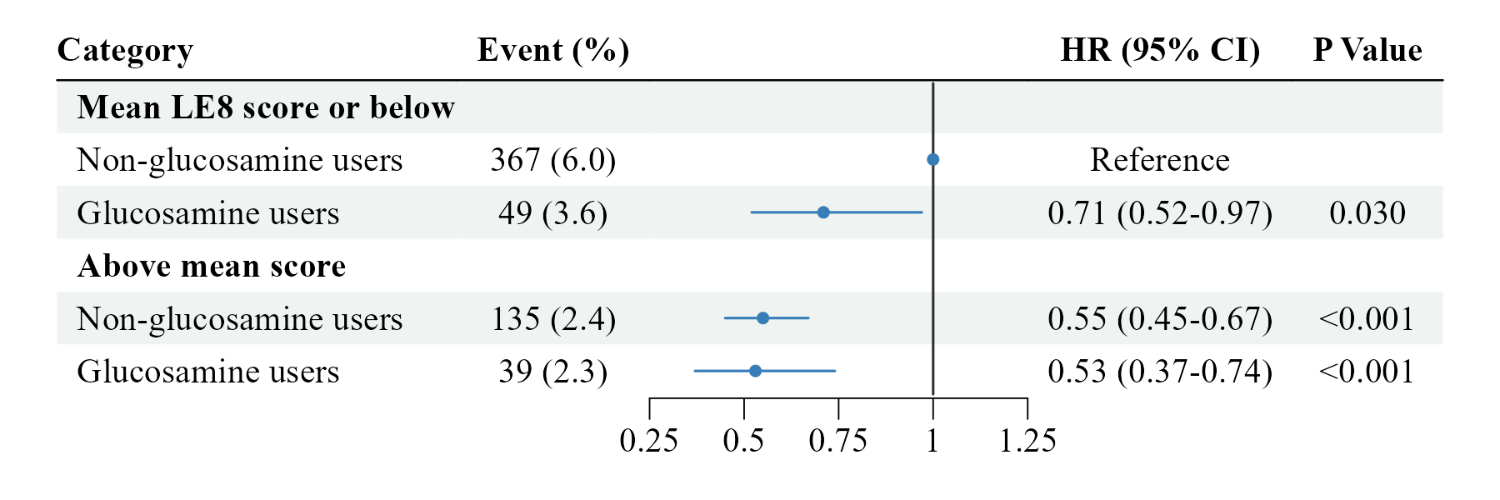


**Joint analysis of the effects of regular use of glucosamine supplements and LE8 score on CVD-related mortality** Participants were classified into four groups based on their regular glucosamine use status (non-users or users) and LE8 score (mean LE8 score of the cohort=64.0 as the cut-off).

Measure of interaction on additive scale (RERI, 95% CI): 1.23 (0.36-2.09).

Measure of interaction on multiplicative scale (95% CI): 1.44 (0.90-2.28), *P* for interaction=0.128.


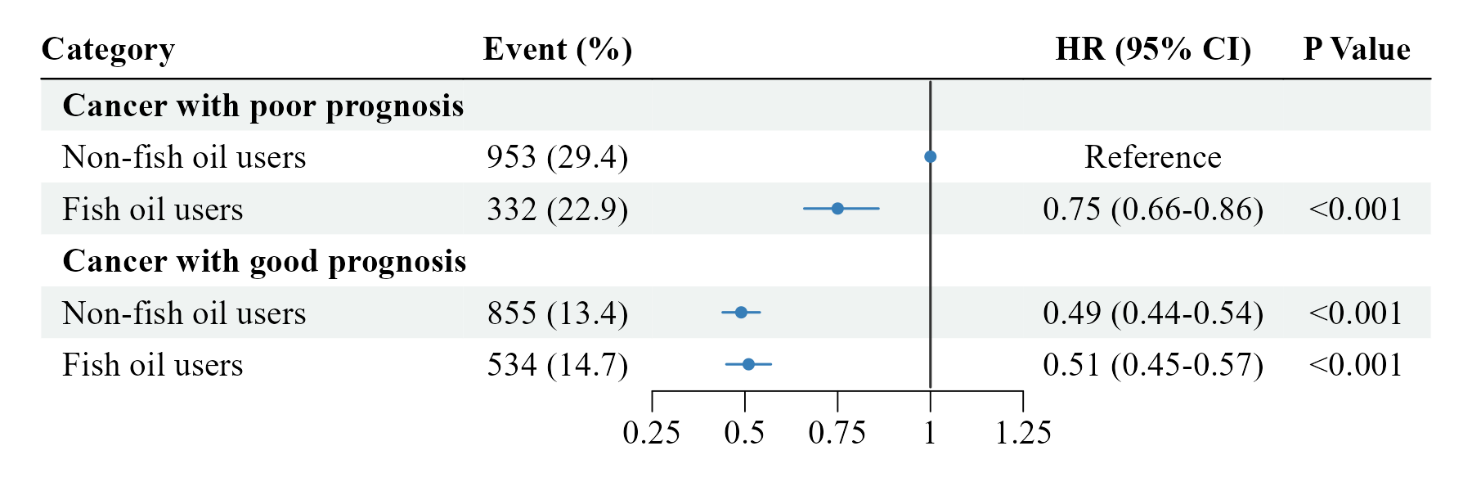


**Joint analysis of the effects of regular use of fish oil supplements and cancer prognosis on all-cause mortality** Participants were classified into four groups based on their regular fish oil use status (non-users or users) and cancer prognosis (categorized based on average prognosis and statistics in the UK Biobank).

Measure of interaction on additive scale (RERI, 95% CI): 1.12 (0.79-1.45).

Measure of interaction on multiplicative scale (95% CI): 1.36 (1.16-1.61), *P* for interaction<0.001.


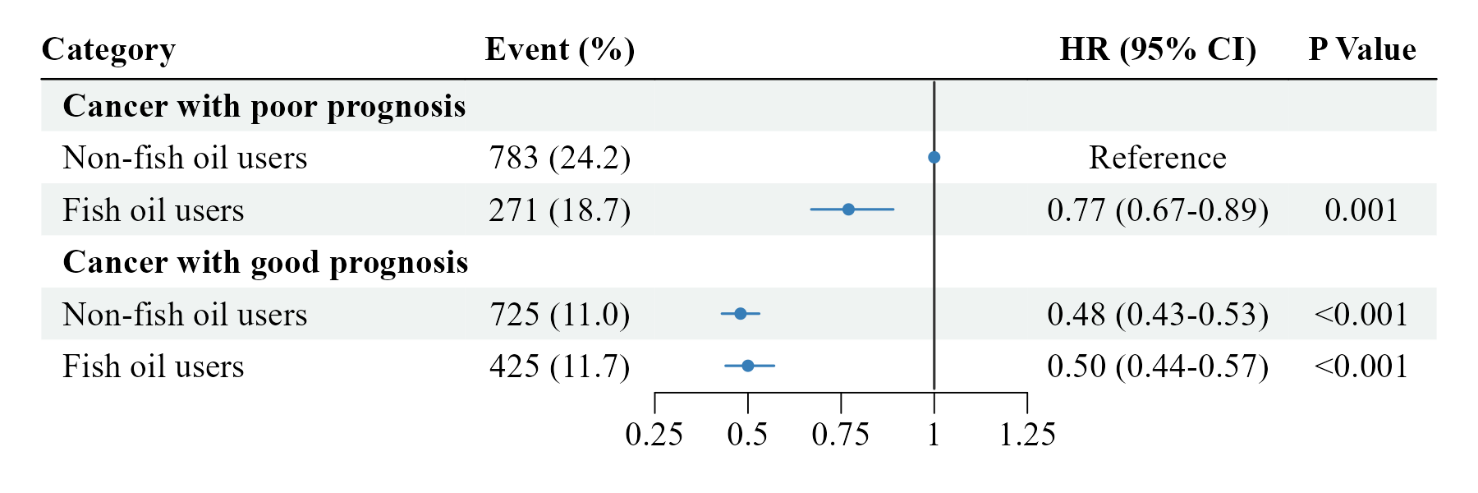


**Joint analysis of the effects of regular use of fish oil supplements and cancer prognosis on cancer mortality** Participants were classified into four groups based on their regular fish oil use status (non-users or users) and cancer prognosis (categorized based on average prognosis and statistics in the UK Biobank).

Measure of interaction on additive scale (RERI, 95% CI): 1.11 (0.75-1.47).

Measure of interaction on multiplicative scale (95% CI): 1.34 (1.12-1.61), *P* for interaction=0.002.


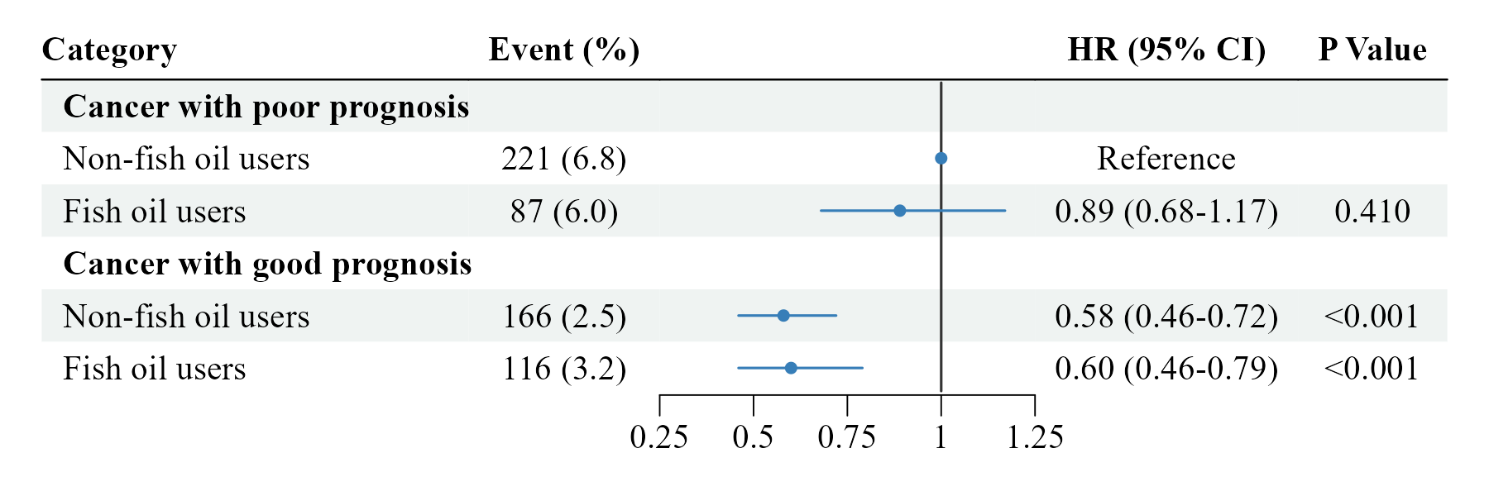


**Joint analysis of the effects of regular use of fish oil supplements and cancer prognosis on CVD-related mortality** Participants were classified into four groups based on their regular fish oil use status (non-users or users) and cancer prognosis (categorized based on average prognosis and statistics in the UK Biobank).

Measure of interaction on additive scale (RERI, 95% CI): 0.98 (0.28-1.67).

Measure of interaction on multiplicative scale (95% CI): 1.34 (0.95-1.89), *P* for interaction=0.097.


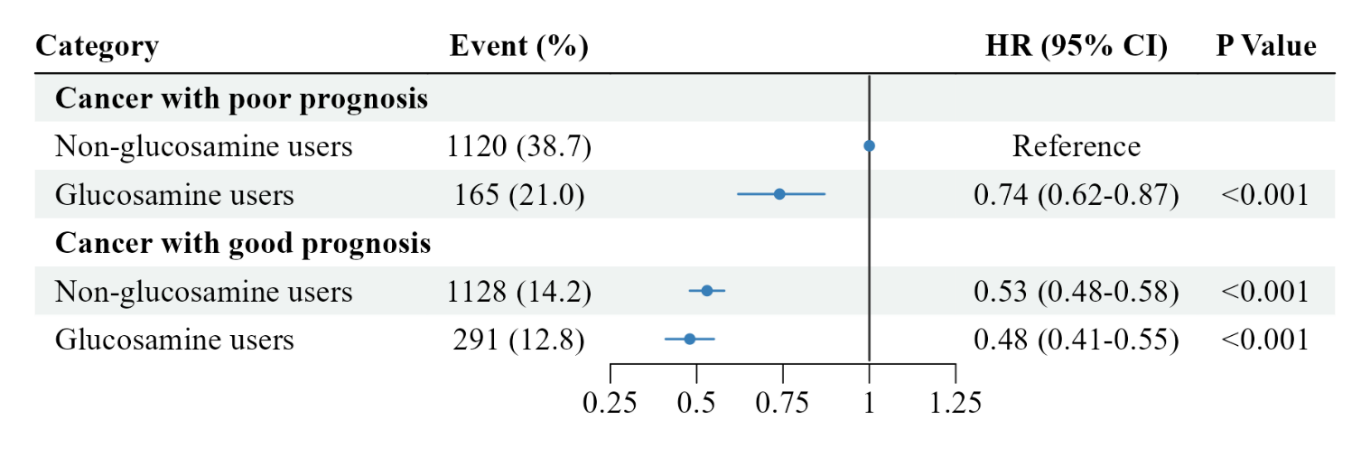


**Joint analysis of the effects of regular use of glucosamine supplements and cancer prognosis on all-cause mortality** Participants were classified into four groups based on their regular glucosamine use status (non-users or users) and cancer prognosis (categorized based on average prognosis and statistics in the UK Biobank).

Measure of interaction on additive scale (RERI, 95% CI): 0.99 (0.61-1.37).

Measure of interaction on multiplicative scale (95% CI): 1.24 (1.01-1.53), *P* for interaction=0.041.


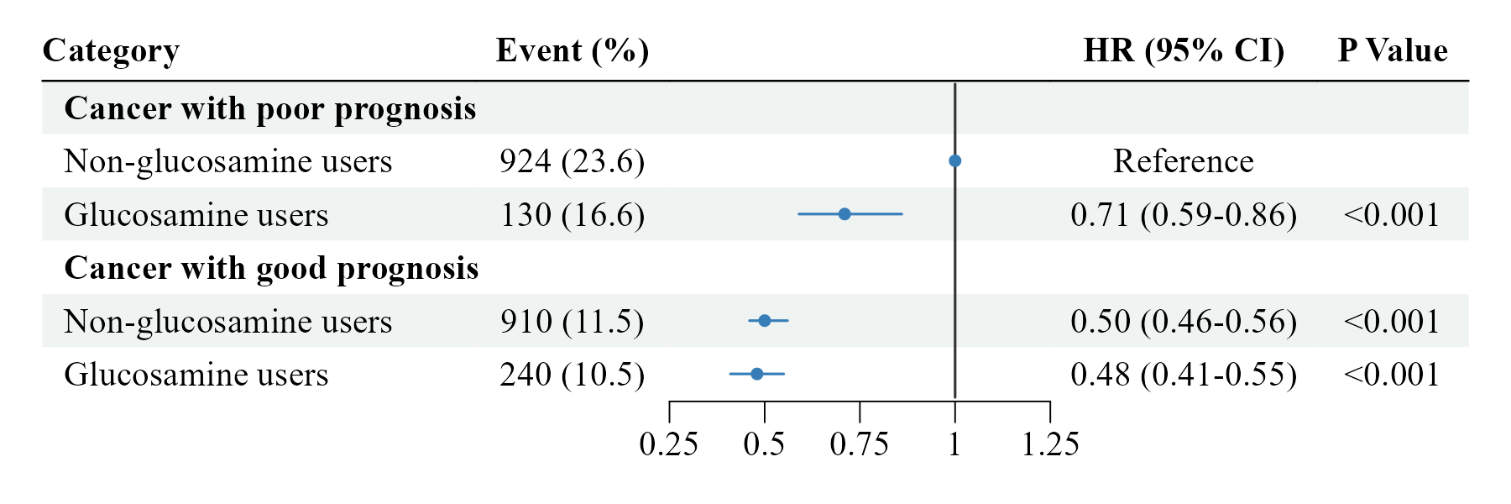


**Joint analysis of the effects of regular use of glucosamine supplements and cancer prognosis on cancer mortality** Participants were classified into four groups based on their regular glucosamine use status (non-users or users) and cancer prognosis (categorized based on average prognosis and statistics in the UK Biobank).

Measure of interaction on additive scale (RERI, 95% CI): 1.15 (0.71-1.59).

Measure of interaction on multiplicative scale (95% CI): 1.34 (1.06-1.69), *P* for interaction=0.014.


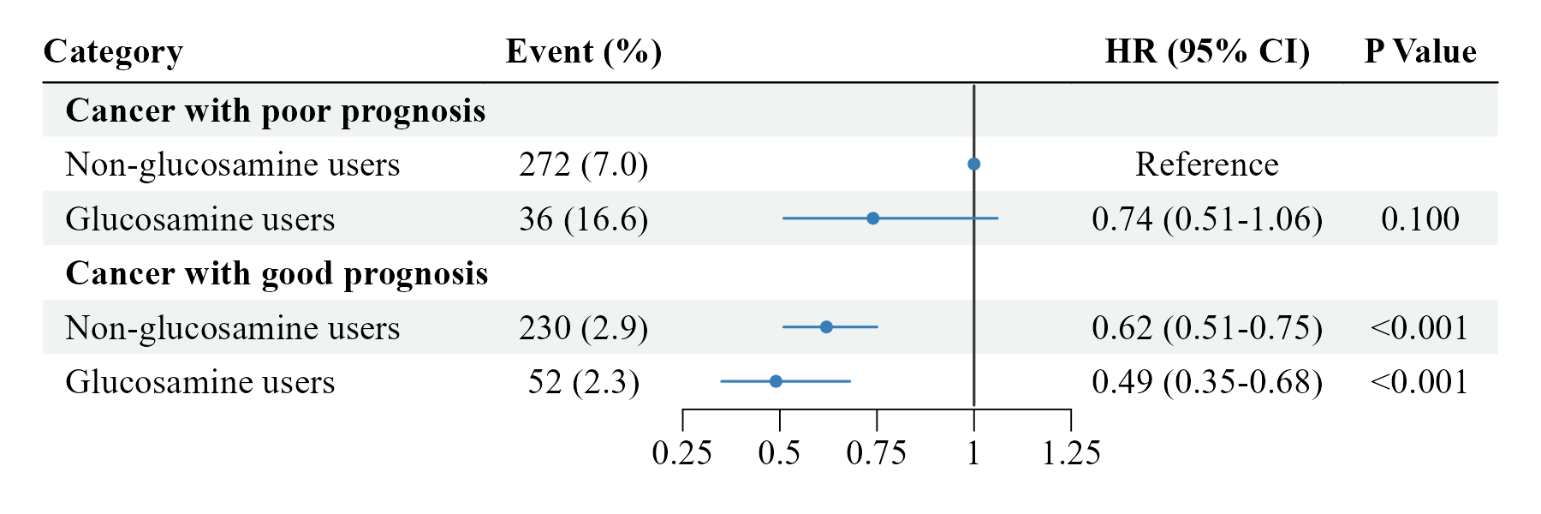


**Joint analysis of the effects of regular use of glucosamine supplements and cancer prognosis on CVD-related mortality** Participants were classified into four groups based on their regular glucosamine use status (non-users or users) and cancer prognosis (categorized based on average prognosis and statistics in the UK Biobank).

Measure of interaction on additive scale (RERI, 95% CI): 0.86 (0.08-1.65).

Measure of interaction on multiplicative scale (95% CI): 1.15 (0.73-1.83), *P* for interaction=0.544
